# The Outcome of Trachomatous Trichiasis Surgery in Ethiopia: Risk Factors for Recurrence

**DOI:** 10.1371/journal.pntd.0002392

**Published:** 2013-08-22

**Authors:** Saul N. Rajak, Esmael Habtamu, Helen A. Weiss, Amir B. Kello, Bayeh Abera, Mulat Zerihun, Teshome Gebre, Clare E. Gilbert, Peng T. Khaw, Paul M. Emerson, Matthew J. Burton

**Affiliations:** 1 The London School of Hygiene and Tropical Medicine, London, United Kingdom; 2 The Carter Center, Addis Abeba, Ethiopia and Atlanta, Georgia, United States of America; 3 Light for The World, Vienna, Austria; 4 Department of Medical Microbiology, College of Medicine, Bahir Dar University, Bahir Dar, Ethiopia; 5 International Trachoma Initiative, The Task Force for Global Health, Addis Ababa, Ethiopia; 6 National Institute for Health Research Biomedical Research Centre, Moorfields Eye Hospital and University College London Institute of Ophthalmology and University College London Partners Academic Health Science Centre, London, United Kingdom; University of California San Francisco, United States of America

## Abstract

**Background:**

Over 1.2 million people are blind from trachomatous trichiasis (TT). Lid rotation surgery is the mainstay of treatment, but recurrence rates can be high. We investigated the outcomes (recurrence rates and other complications) of posterior lamellar tarsal rotation (PLTR) surgery, one of the two most widely practised TT procedures in endemic settings.

**Methodology/Principal Findings:**

We conducted a two-year follow-up study of 1300 participants who had PLTR surgery, conducted by one of five TT nurse surgeons. None had previously undergone TT surgery. All participants received a detailed trachoma eye examination at baseline and 6, 12, 18 and 24 months post-operatively. The study investigated the recurrence rates, other complications and factors associated with recurrence. Recurrence occurred in 207/635 (32.6%) and 108/641 (16.9%) of participants with pre-operative major (>5 trichiatic lashes) and minor (<5 lashes) TT respectively. Of the 315 recurrences, 42/315 (3.3% overall) had >5 lashes (major recurrence). Recurrence was greatest in the first six months after surgery: 172 cases (55%) occurring in this period. Recurrence was associated with major TT pre-operatively (OR 2.39, 95% CI 1.83–3.11), pre-operative entropic lashes compared to misdirected/metaplastic lashes (OR 1.99, 95% CI 1.23–3.20), age over 40 years (OR 1.59, 95% CI 1.14–2.20) and specific surgeons (surgeon recurrence risk range: 18%–53%). Granuloma occurred in 69 (5.7%) and notching in 156 (13.0%).

**Conclusions/Significance:**

Risk of recurrence is high despite high volume, highly trained surgeons. However, the vast majority are minor recurrences, which may not have significant corneal or visual consequences. Inter-surgeon variation in recurrence is concerning; surgical technique, training and immediate post-operative lid position require further investigation.

## Author Summary

Trachoma is the most common infectious cause of blindness worldwide. It causes trichiasis (inturning of the eyelashes to touch the eye), which can cause visual loss. Trachomatous trichiasis (TT) affects over eight million people, 1.2 of whom live in Ethiopia – the most affected country worldwide. Surgery is the mainstay of treatment for TT. However, results of surgery in the field are often very mixed. We investigated the surgical outcomes of one of the two most widely used surgical techniques (posterior lamellar rotation), in 1300 individuals in the Amhara Region of Ethiopia. We found that recurrence occurred frequently: 315/1276 (24.7%) participants. However, recurrence was rarely severe (greater than 5 lashes): 42 participants (3.3%). Recurrence occurred much more frequently in participants who had severe pre-operative disease and with specific surgeons. The high recurrence rates and inter-surgeon variation is concerning. Further research will be required to investigate factors such as surgical technique, surgeon training and immediate post-operative lid position, in order to improve surgical outcomes.

## Introduction

Blindness from trachoma is the end result of progressive scarring of the conjunctiva driven by *Chlamydia trachomatis*. The major risk factor of blinding corneal opacification (CO) is trichiasis (TT), the in turning of the eyelashes. TT traumatises the delicate epithelium of the cornea, rendering it vulnerable to secondary infection. TT encompasses a range of eyelid and eyelash abnormalities from a few peripheral in turned lashes to the entire upper eyelid pulled inwards by scarring (entropion). TT can also occur without entropion, from metaplastic or misdirected lashes [1]. Recent global estimate suggested that in 2008 there were 8.2 million people living with trichiasis. Surgical treatment for TT is a key component of the SAFE strategy for trachoma control, directly reducing the risk of blindness [2].

Several different surgical procedures have been used to correct upper lid TT, some of which have been compared in randomized trials [3]–[6]. The technique of Bilamellar Tarsal Rotation (BLTR) has the lowest recurrence risk. However, the widely used Posterior Lamellar Tarsal Rotation (PLTR or ‘Trabut’ procedure) was not included in these comparisons. The World Health Organisation (WHO) advocates either BLTR or PLTR surgery for TT [7]. Both procedures involve a horizontal tarsotomy combined with everting sutures to rotate the inferior portion of the upper lid outwards [3].

TT surgery can prevent or reduce progression of corneal opacity, improve vision and relieve pain [6], [8], [9]. However, surgical outcomes are variable. Most studies of post-operative TT recurrence reports rates of 20%–40% at one-year, ranging from 7.4% at one year to 62% at three years [5], [6], [8], [10]–[22]. Recurrence is generally subdivided into early (before six months) and late (after six months). Early recurrence is probably attributable to a number of factors including the severity of the preoperative disease, the type and quality of the surgery, and post-operative wound healing events [8], [23], [24]. Substantial inter-surgeon variation of TT recurrence rates has been reported [8], [23]. After six months there is a steady accumulation of recurrence that probably results from progressive scarring disease [8], [22].

Serious surgical complications are rare in TT surgery. However, complications such as granuloma and lid contour abnormalities (notching) occur relatively frequently [23]. Granulomas are pedunculated masses of inflammatory tissue ranging in size from a few millimetres to over a centimetre. Larger granulomas can obscure the visual axis, and all except the smallest require surgical removal. The reported frequency of granulomas ranges from 0% to 14% [6], [10], [11], [16], [23], [25]–[28]. Lid notching, a focal overcorrection of the lid caused by irregular suture tension or an irregular tarsal incision is cosmetically unsatisfactory and may be associated with lagophthalmos, putting the cornea at risk [29].

During the course of two recently reported randomised controlled trials conducted in Ethiopia we recruited 1300 individuals with the full spectrum of TT type and severity, who received PLTR surgery with silk sutures, and were followed up for two year [30], [31]. This represents the largest data set to date on the results of PLTR surgery (recurrence risks, vision and other outcomes), and provides an opportunity to investigate outcomes in relation to the type and position of the trichiatic lashes.

## Methods

### Ethical approval

The National Health Research Ethics Review Committee of the Ethiopian Ministry of Science and Technology, the London School of Hygiene & Tropical Medicine Ethics Committee and the Emory University Institutional Review Board approved this study. Informed consent was taken at the time of enrolment. The research adhered to the tenets of The Declaration of Helsinki. All participants gave written informed consent to take part in the study.

### Study design and participants

Two previously reported randomised controlled trials of the management of TT were conducted in Ethiopia from 2008 to 2010 [30], [31]. Each trial recruited 1300 individuals aged 18 years or older with previously unoperated TT: in each trial one arm comprised participants undergoing TT surgery with silk sutures. For the purpose of these studies, TT was defined as one or more lashes touching the eye or clear evidence of epilation (broken/re-growing lashes), without another obvious cause for the trichiasis, such as trauma, malignancy, involutional changes or severe blepharitis. Exclusion criteria were previous eyelid surgery and self-reported pregnancy.

Participants presented during TT surgical treatment campaigns in rural villages in the West Gojjam Zone, Amhara Regional State, which were advertised in local markets, churches and schools. Additionally, health extension workers from every sub-district (kebele) across West Gojjam were trained to recognize trichiasis. They visited each village in their kebele to identify potential participants.

In the first trial, individuals with major TT (>5 lashes touching the eye) were randomly allocated to PLTR surgery with either silk or polyglactan (vicryl) sutures. In the second trial individuals with minor TT (<6 lashes touching) were randomly allocated to either PLTR surgery with silk sutures or repeated epilation. In individuals with bilateral TT, one eye was randomly designated (sequentially selected from a blocked randomly generated list of right and left eyes) as the *study eye* and included in the analysis, although both eyes were treated. In both trials, participants were allocated to surgeons sequentially. Surgeons were not permitted to select specific participants and participants were not allocated according to severity.

The group described in this report is comprised of all the individuals who were randomly allocated to the PLTR with silk suture arms of the two studies. They represent the full spectrum of TT disease (both major and minor TT) and received exactly the same surgical intervention performed by the same group of surgeons.

### Clinical assessment

Participants were examined immediately before surgery and again at 6, 12, 18 and 24 months. The methods used have been described in detail [30], [31]. Briefly, LogMAR visual acuity was measured in each eye. Participants' weight and height were measured in order to calculate the body mass index (BMI). Both eyes were examined for signs of trachoma using 2.5× magnification loupes and a bright torch. Baseline, one-year and two-year examinations were by a single ophthalmologist (SNR) and the six and 18-month examinations were by a single ophthalmic nurse (EH). The examiners were standardised to each other. Lashes touching the eye were counted (‘lash burden’) and sub-divided into the part of the eye contacted when looking straight ahead (corneal or peripheral (lateral or medial conjunctiva) and subdivided by the type of trichiatic lash (entropic, misdirected or metaplastic). Clinical evidence of epilation was defined as the presence of broken or newly growing lashes, or areas of absent lashes. In the absence of epilation, eyes with <6 trichiatic lashes were designated as minor TT and those with >5 lashes as major TT. In the presence of epilation, a clinical judgement was made of the number of epilated lashes, by assessing regrowing lash stubs that were pointing towards the globe; if the total trichiatic lashes+epilated lashes was <6, the lid was designated as having minor TT and >5 as major TT. Upper lid entropion was graded using a previously described system [1]. Corneal scarring was classified based on a modified WHO FPC grading system [32], [33]. Corneal opacity was graded in the field and with high-resolution digital photographs (Nikon D300, Nikon 105 mm macro lens). The eyelid was everted and the location of the muco-cutaneous junction (MCJ) graded [1]. Following surgery the presence/absence of notching and granuloma were noted. Notching was defined as central overcorrection of the lid such as to cause a clear deviation in contour of the lid margin. This would correspond to either moderate or severe lid contour abnormalities in a recently published grading system [34].

### Interventions

Surgery was performed under local anaesthesia using the technique previously described [30], [31]. Post-operatively, the operated eye was padded for a day and then tetracycline eye ointment was self-administered twice a day for two weeks. Five nurses, who had previously been trained in and were regularly performing PLTR surgery, performed the surgery. They were selected as the best surgeons from a larger group of 10, during a two-day standardisation workshop. The PLTR techniques of the five nurses were carefully observed and standardised to ensure that all performed the operation in the same way. The intra-operative quality of surgery was periodically reviewed during the course of the trials. Participants were seen at 7–10 days post-operatively, at which point silk sutures were removed. The presence of trichiasis and other complications were noted and treated as needed. Any individual who had five or more lashes at any follow-up examination was offered repeat surgery to be performed by a senior surgeon, within a few weeks of the follow-up assessment. Individuals in whom other ophthalmic pathology (e.g. cataract) was detected were referred to the regional ophthalmic services.

### Outcome measures

The primary outcome measure was trichiasis recurrence defined as either (1) one or more eyelashes touching the eye or (2) clinical evidence of epilation. Secondary outcome measures were surgical complications, entropion and conjunctivalisation.

### Statistical methods

Data were double entered into an Access (Microsoft) database and transferred to Stata 11 (StataCorp, College Station, TX). For participants who had bilateral surgery only the randomly designated ‘study eye’ was included in the analysis. The cumulative incidence of failure in each six-month block of follow-up was calculated using the Kaplan-Meier method. The associations of binary outcomes with exposures were assessed using logistic regression to estimate odds ratios (OR) and 95% confidence intervals (CI). Variables associated with the outcome on univariate analyses (p<0.2) were retained in the multivariable model. The p-values for the association between categorical variables and specific outcomes were calculated using likelihood ratios. For visual acuities of counting fingers or less, logMAR values were attributed: counting fingers: 2.0, hand movements: 2.5, perception of light: 3.0, no perception of light: 3.5.

## Results

We recruited 1300 individuals with previously unoperated trichiasis in at least one eye. No individuals refused participation. Recruitment took place between March and July 2008. The follow-up assessments were conducted at the following times: 6-month, August to December 2008; 12-month, February to July 2009; 18-months, August to December 2009; 24-months, January 2010 to May 2010. The final follow-up was conducted two months ahead of schedule because of the Ethiopian general election (May 2010), during which time follow-up would not have been possible. Almost all individuals (98.1%) were reviewed on at least one occasion.

Baseline characteristics are shown in Table 1; 650 eyes had major TT and 650 had minor TT. All participants were from the Amhara region of Ethiopia. The logMAR visual acuity was 0.3 (6/12 Snellen equivalent) or less in 853/1289 (66%) individuals it was possible to test. Of those with major TT, 359 had less than five lashes, but had evidence of epilation consistent with the eye having more than five trichiatic lashes. The majority of the participants were female (78.2% of those with major TT, 68.9% of those with minor TT: p = 0.005). Corneal opacity was present in 768 (59.9%) individuals of whom 419 (32% of all study eyes) had opacity within the central 4 mm of the cornea.

**Table 1:** Demographic and pre-operative clinical characteristics of participants, sub-divided by major and minor trichiasis.

	Minor TT (<6 lashes)		Major TT (>5 lashes)	
Eyes	650		650	
Sex (female)	448	(68.9%)	508	(78.2%)
Age				
18–30	69	(10.6%)	48	(7.4%)
30–39	113	(17.4%)	123	(18.9%)
40–49	156	(24.0%)	186	(28.6%)
50–59	160	(24.6%)	167	(25.7%)
60–69	112	(17.2%)	93	(14.3%)
70+	40	(6.2%)	33	(5.1%)
mean (sd, 95% C.I.)	49.9	(14.4, 48.8–51.0)	49.7	(12.9, 48.7–50.7)
Illiterate	581	(89.4%)	617	(94.9%)
BMI, mean (sd, 5% CI)	19.9	(2.3, 19.7–20.1)	20.1	(2.5, 19.9–20.3)
Right eye*	319	(49.1%)	321	(49.4%)
Best corrected logMAR VA in study eye				
−0.2–0.3	243	(37.7%)	193	(30.0%)
0.3–0.7	261	(40.5%)	248	(38.5%)
0.7–1.1	83	(12.9%)	104	(16.2%)
1.1–2.0	19	(2.9%)	29	(4.5%)
CF/HM/PL	30	(4.6%)	62	(9.6%)
NPL	9	(1.4%)	8	(1.2%)
Not measurable†	5		6	
Entropion grade				
0	276	(42.5%)	51	(7.9%)
1	258	(39.7%)	159	(24.5%)
2	113	(17.4%)	242	(37.2%)
3	3	(0.5%)	119	(18.3%)
4	0	(0.0%)	79	(12.2%)
Trichiasis (number of lashes) (median: 1)				
None - epilating	124	(19.1%)	106*	(16.3%)
1–5	526	(80.9%)	253*	(38.9%)
6–9	0	(0.0%)	152	(23.4%)
10–19	0	(0.0%)	89	(13.7%)
20+	0	(0.0%)	50	(7.7%)
Lash location				
None – epilating	124	(19.1%)	106	(16.3%)
Corneal +/− peripheral	462	(71.1%)	524	(80.6%)
Peripheral only	64	(9.9%)	20	(3.1%)
Lash type				
None (epilating)	124	(19.1%)	106	(16.3%)
Metaplastic only	299	(46.0%)	206	(31.7%)
Misdirected only	165	(25.4%)	83	(12.8%)
Metaplastic and misdirected	59	(9.1%)	67	(10.3%)
Entropic +/− aberrant	3	(0.5%)	188	(28.9%)
Lower lid TT				
Present	70	(10.8%)	88	(13.5%)
Corneal opacity				
None (C0)	345	(53.1%)	187	(28.8%)
Peripheral (C1)	168	(25.9%)	181	(27.9%)
Off centre faint (C2a)	76	(11.7%)	147	(22.6%)
Off centre dense (C2b)	9	(1.4%)	26	(4.0%)
Central faint (C2c)	30	(4.6%)	63	(9.7%)
Central dense (C2d)	11	(1.7%)	18	(2.8%)
Total central dense (C3)	10	(1.5%)	22	(3.4%)
Phthisis (C4)	1	(0.2%)	6	(0.9%)
Papillary inflammation				
None (P0)	52	(8.0%)	52	(8.0%)
Mild (P1)	248	(38.2%)	183	(28.2%)
Moderate (P2)	297	(45.7%)	312	(48.1%)
Severe (P3)	53	(8.2%)	102	(15.7%)
Conjunctival scarring				
None (C0)	3	(0.5%)	0	(0.0%)
Mild (C1)	60	(9.2%)	18	(2.8%)
Moderate (C2)	508	(78.2%)	400	(61.6%)
Severe (C3)	79	(12.2%)	231	(35.6%)
Conjunctivalisation Grade				
0	7	(1.1%)	2	(0.3%)
1	18	(2.8%)	5	(0.8%)
2	165	(25.4%)	96	(14.8%)
3	460	(70.8%)	545	(84.1%)
Lagophthalmos	10	(1.5%)	20	(3.1%)

* Despite having less than 5 lashes actually touching the globe at the time of examination, these individuals had clinical evidence of epilation of trichiatic lashes, such that the total of trichiatic lashes would have been greater than 5 had they not epilated.

† Patient unable to co-operate with visual acuity measurement.

### Recurrent trichiasis

**Figure 1:**
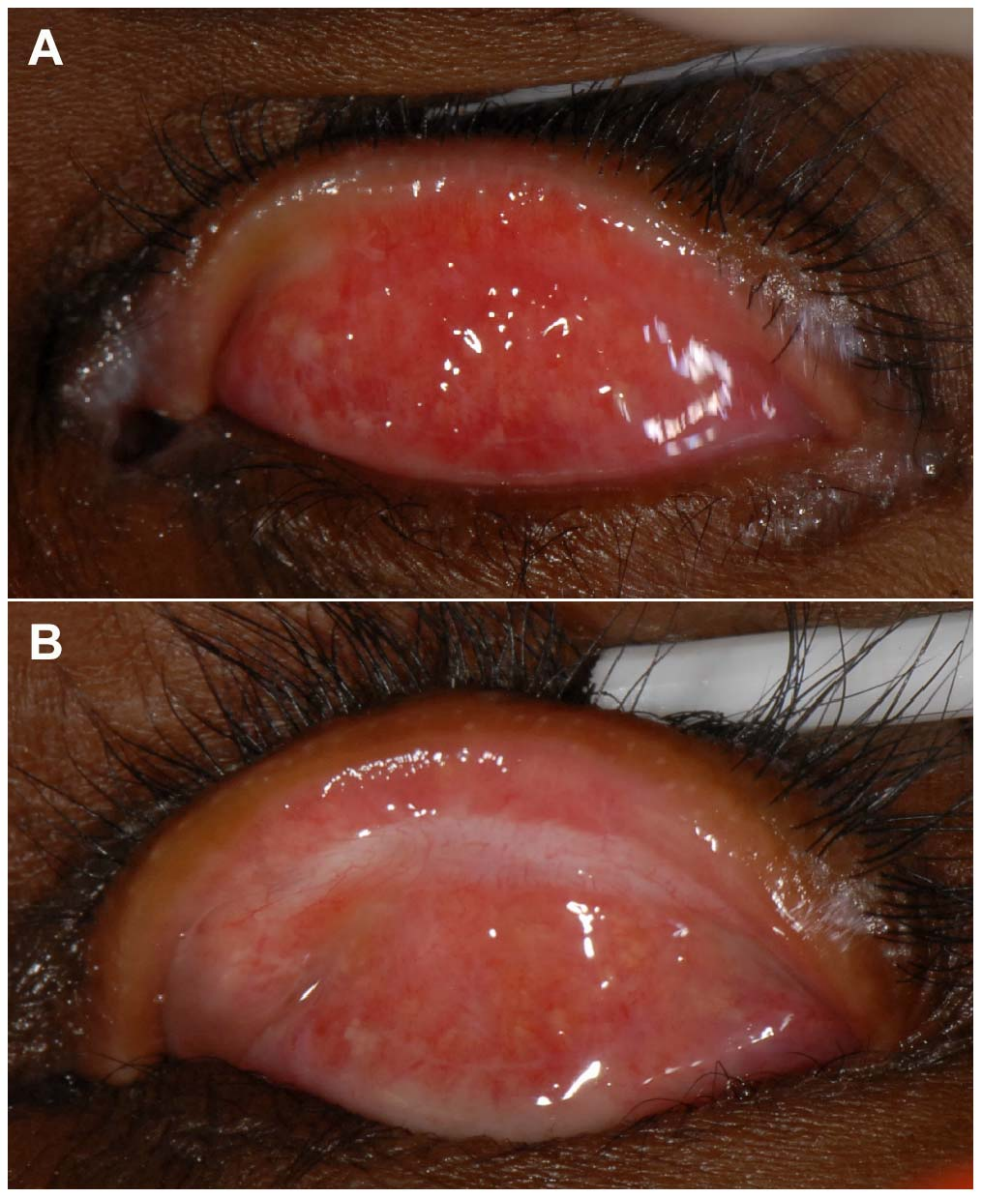
Conjunctivalisation of the lid margin. 1a: The lid margin at baseline showing marked conjunctivalisation; the Meibomian gland orifices are completely surrounded by conjunctival-type surface. 1b: The lid margin of the same participant at the two-year follow-up; the conjunctival surface appears to have receded and the Meibomian gland orifices are surrounded by skin with a normal appearance.

Overall, recurrence occurred in 315/1276 (24.7%) study eyes. Recurrence was more frequent in participants with pre-operative major TT (32.6%) compared to minor TT (16.9%): OR 2.39, 95%CI 1.83–3.11, p<0.005 (Figure 1 and Table 2). This association was found at each follow-up (Table 2). Within both TT groups, the risk of recurrence was much higher during the first six-month period compared to all subsequent periods (p<0.0001; Figure 1 and Table 2), with 58.0% and 48.1% of recurrences accruing during this initial period in major TT and minor TT participants, respectively. Thirty-eight participants had recurrence at the 6 months follow-up, but not at any subsequent timepoint, of whom ten had repeat TT surgery.

**Table 2:** Risk of trichiasis recurrence and severity, subdivided by baseline severity.

	Baseline TT Severity					
	Minor TT		Major TT		Total	
**a) Number of Incident Recurrence Cases at each time-point (months)**						
6	52/615	(8.5%)	120/587	(20.4%)	172/1202	(14.3%)
12	23/622	(3.7%)	37/612	(6.1%)	60/1234	(4.9%)
18	22/620	(3.6%)	34/622	(5.5%)	56/1242	(4.5%)
24	11/614	(1.8%)	16/619	(2.6%)	27/1233	(2.2%)
**Total**	**108/641** *	**(16.9%)**	**207/635** *	**(32.6%)**	**315/1276** *	**(24.7%)**
**b) Recurrence severity**						
None	533	(83.2%)	428	(67.4%)	961	(75.3%)
Minor	104	(16.2%)	169	(26.6%)	273	(21.4%)
Major (at 1 or more follow-ups)	4	(0.6%)	38	(6.0%)	42	(3.3%)
**Total**	**641**		**635**		**1276**	
**c) Recurrence risks by eye**						
Right	49/314	(15.6%)	102/316	(32.3%)	151/630	(24.0%)
Left	59/327	(18.0%)	105/319	(32.9%)	164/646	(25.4%)
**Total**	**108**		**207**		**315**	
**d) Change in lash burden between baseline and 24 months in participants with recurrence**						
>5 more lashes at 24 m	0	(0.0%)	2	(1.0%)	2	(0.7%)
1–5 more lashes at 24 m	2	(1.9%)	12	(6.1%)	14	(4.6%)
Same number lashes at 24 m	26	(24.3%)	35	(17.7%)	61	(20.0%)
1–5 fewer lashes at 24 m	79	(73.8%)	66	(33.3%)	145	(47.5%)
6–20 fewer lashes at 24 m	0	(0.0%)	69	(34.9%)	69	(22.6%)
>20 fewer lashes at 24 m	0	(0.0%)	14	(7.1%)	14	(4.6)
**Total**	**107**	**(100%)**	**198**	**(100%)**	**305**	**(100%)**
**e) Recurrence risks by baseline lash type**						
No lashes (epilating)	11/123	(8.9%)	34/101	(33.7%)	45/224	(20.1%)
Metaplastic lashes only	54/293	(18.4%)	64/200	(32.0%)	118/493	(23.9%)
Misdirected lashes only	27/164	(16.5%)	16/83	(19.3%)	43/247	(17.4%)
Aberrant lashes†	15/58	(25.9%)	14/65	(21.5%)	29/123	(23.6%)
Entropic +/− aberrant lashes	1/3	(33.3%)	79/186	(42.5%)	80/189	(42.3%)

* Number of individuals seen on at least one follow-up.

† Aberrant lashes = either metaplastic or misdirected.

(a) Incident recurrence at each time-point. (b) Severity of the recurrence. (c) Recurrence rates by eye. (d) Change in the number of lashes between baseline and 24 months. (e) Recurrence by type of lash at baseline.

Overall, there was a significant reduction in mean lash burden between baseline (4.66 lashes) and 24 months (0.29 lashes; t-test p<0.0005) (Table 2). Amongst people with recurrence who were examined at 24 months, 14/198 (7.1%) of those with baseline major TT had more trichiasis at 24 months compared to baseline, compared with 2/107 (1.9%) of those with baseline minor TT (Table 2). There were similar risks of recurrence in right and left eyes in both groups (Table 2).

Individual surgeon's recurrence rates ranged from 17.7% to 52.6%. The pre-operative severity of cases operated by the different surgeons varied to a degree (X^2^: p = 0.035), as did their post-operative under-correction rates (X^2^ = 0.003) (Table 3). Their risk of recurrence was not affected by the variation in case mix (Table 4). There was no significant difference between the recurrence rate in the first 20 surgeries conducted by each surgeon (32/100, 32%) and the last 20 surgeries (29/100, 29%, p = 0.42).

**Table 3:** Baseline severity of cases operated (case mix), under-correction and overall trichiasis recurrence rates by surgeon.

	Surgeon ID										Total	
	1		2		3		4		5			
**Baseline TT Severity**												
Minor	186	(49.5%)	149	(52.7%)	167	(55.1%)	118	(45.4%)	30	(38.5%)	650	(50%)
Major	190	(50.5%)	134	(47.4%)	136	(44.9%)	142	(54.6%)	48	(61.5%)	650	(50%)
Total	376		283		303		260		78		1300	
**TT at 7–10 days post-operatively** *												
	11	(2.9%)	9	(3.2%)	5	(1.7%)	7	(2.7%)	8	(10.3%)	40	(3.1%)
**Recurrence during the 24 months of follow-up** §												
Yes	75	(20.3%)	88	(32.0%)	67	(22.3%)	45	(17.7%)	40	(52.6%)	315	(24.7%)
Total Seen	370		275		301		254		76		1276	

* Denominator: total number of patients operated by that surgeon.

§ Denominator: participants seen on at least one follow up occasion.

**Table 4:** Univariable analysis and multivariable logistic regression model for baseline associations with any TT recurrence.

Variable	Univariable Analysis			Multivariable Analysis		
	OR	95% CI	p Value	OR	95% CI	p Value
Female	1.11	0.83–1.49	0.47			
Age>40	1.65	1.21–2.24	0.002	1.59	1.14–2.20	0.006
Literate	0.79	0.48–1.29	0.35			
BMI<18	0.70	0.51–0.95	0.024	0.79	0.56–1.09	0.15
Major TT at baseline	2.39	1.83–3.11	<0.001	1.93	1.43–2.60	<0.001
Eye (Right)	1.08	0.84–1.39	0.56			
Baseline entropion severity*	1.35	1.21–1.51	<0.001			
Surgeon†			<0.001			<0.001
1	1.18	0.78–1.78		1.23	0.81–1.88	
2	2.19	1.45–3.29		2.46	1.60–3.77	
3	1.33	0.87–2.03		1.49	0.9762.30	
5	5.16	2.97–8.98		5.18	2.90–9.26	
Baseline lash type‡			<0.001			<0.001
Metaplastic only	1.25	0.85–1.84		1.27	0.85–1.90	
Misdirected only	0.84	0.53–1.33		0.89	0.55–1.43	
Metaplastic and misdirected	1.23	0.72–2.08		1.10	0.63–1.92	
Entropic +/− aberrant	2.92	1.89–4.51		1.99	1.23–3.20	
Purulent discharge at baseline	1.86	1.17–2.95	0.009	1.21	0.73–2.02	0.46
Mucus discharge at baseline	1.39	0.63–3.06	0.42			
Baseline Inflammation (P2/P3)	1.37	1.06–1.79	0.018	1.23	0.93–1.63	0.15

* Entropion treated as a linear variable (LR test for linearity: p = 0.07).

Entropion dropped from multivariable model as strongly linked to baseline lash type.

† Compared to surgeon 4 who had the lowest recurrence rate. Likelihood ratio used to calculate p value for association between surgeon and recurrent TT.

‡ Compared to group with all trichiatic lashes epilated. Likelihood ratio used to calculate p value for association between lash type and recurrent TT.

Multivariable logistic regression modelling identified increased baseline TT severity, the presence of entropic lashes (compared with misdirected or metaplastic lashes without entropion), specific surgeons (No. 2 and No. 5) and older participant age as independent risk factors for recurrence (Table 4).

### Surgical complications

Early (noted by the 7–10 day suture removal follow-up) and later (seen at any subsequent follow-up) post-operative complications and their association with recurrent trichiasis are presented in Table 5. Over three-quarters (23/30, 77%) of individuals noted to have trichiasis at the 7–10 day follow-up had recurrent trichiasis at a subsequent follow-up (OR: 10.3, 95% C.I.: 4.33–24.23, p<0.001) (Table 6). Post-operative granuloma (OR: 0.39, 95% C.I.: 0.19–0.83, p = 0.014) and notching (OR: 0.44, 95% C.I.: 0.28–0.72, p = 0.001) were both significantly associated with lower recurrence rates (Table 6).

**Table 5:** Frequency and risk of post-operative complications, stratified by baseline disease severity. Trichiasis recurrence rates in eyes with the specific complication.

	Baseline TT Severity					
	Minor TT		Major TT		Total	
**Intra-operative**						
Bleeding	11	(1.7%)	6	(0.9%)	17	(1.3%)
**Early (by 7–10 days)**						
TT recurrence at 7–10 days	5	(0.8%)	24	(3.8%)	29	(2.3%)
Overcorrection	2	(0.3%)	4	(0.6%)	6	(0.5%)
Infection/erythematous swelling/conjunctivitis	3	(0.5%)	6	(0.9%)	10	(0.8%)
Infection & undercorrection	1	(0.2%)	0	(0.0%)	1	(0.1%)
Bleeding	3	(0.5%)	1	(0.2%)	4	(0.3%)
**Late (6,12,18 or 24 month)**						
Granuloma	18	(2.9%)	51	(8.7%)	69	(5.7%)
Notching	86	(14.0%)	70	(11.9%)	156	(13.0%)
Residual suture fragments	7	(1.1%)	15	(2.6%)	22	(1.8%)
Lid abscess	1	(0.2%)	0	(0.0%)	1	(0.1%)

**Table 6:** Univariable and multivariable associations between recurrent TT and intra- and post-operative complications.

Variable	Univariable Analysis			Multivariable Analysis		
	OR	95% CI	p Value	OR	95% CI	p Value
Intra-operative haemorrhage	0.65	0.18–2.27	0.50			
Trichiasis at 7–10 days	10.25	4.33–24.23	<0.001	10.44	4.20–25.95	<0.001
7–10 day overcorrection	1.63	0.30–8.95	0.57			
7–10 day infection/swelling/conjunctivitis	1.63	0.41–6.56	0.49			
Granuloma	0.39	0.19–0.83	0.014	0.31	0.13–0.74	0.008
Notching	0.44	0.28–0.72	0.001	0.52	0.32–0.85	0.009
Residual suture fragment(s)	1.47	0.59–3.63	0.409			
Inflammation (P2/P3) at 12 months	1.70	1.14–2.54	0.01	1.64	1.06–2.53	0.027

Univariate and multivariable associations for developing a granuloma and lid notching are shown in Tables 7. Surgeons (No. 1 and No. 4) who had the lowest recurrence rates also had significantly higher rates of granuloma and notching. There was no association between granuloma formation and visible suture fragments being left in the lid (X^2^: p = 0.495), gender (X^2^: p = 0.239) or younger (<41 years) age (X^2^: p = 0.41)

**Table 7:** Univariable and multivariable intra and post-operative associations for granuloma and notching.

Variable	Granuloma						Notching					
	Univariable analysis			Multivariable analysis			Univariable analysis			Multivariable analysis		
	OR	95% CI	p Value	OR	95% CI	p Value	OR	95% CI	p Value	OR	95% CI	p Value
Recurrence	0.39	0.19–0.83	0.014	0.33	0.15–0.71	0.005	0.39	0.19–0.83	0.014	0.49	0.30–0.81	0.005
>5 lashes at baseline	3.16	1.82–5.47	<0.001	4.07	2.16–7.67	<0.001	0.83	0.59–1.17	0.29			
Baseline entropion severity	1.17	0.95–1.44	0.15	0.86	0.66–1.11	0.25	0.90	0.77–1.05	0.18	0.96	0.82–1.13	0.35
Surgeon*			0.0267			<0.001			<0.001			0.002
1	2.23	1.06–4.71		0.62	0.30–1.27		2.20	1.28–3.79		2.02	1.17–3.49	
2	1.31	0.56–3.09		0.44	0.20–0.93		1.0	n/a		1.0		
3	1.0	n/a		1.0	n/a		1.76	0.99–3.13		1.62	0.91–2.91	
4	2.42	1.11–5.27		0.97	0.52–1.81		2.71	1.55–4.74		2.47	1.40–4.35	
5	0.41	0.05–3.29		0.18	0.023–1.39		0.37	0.084–1.63		0.41	0.09–1.83	
Inflammation at baseline (P2/P3)	2.14	1.29–3.57	0.003	2.23	1.33–3.78	0.003	0.67	0.47–0.94	0.022	0.75	0.53–1.07	
Female	0.73	0.44–1.23	0.24				0.79	0.55–1.14	0.21			
Age>40 yrs	0.80	0.47–1.35	0.41				2.06	1.32–3.20	0.001	1.99	1.26–3.12	0.003
BMI>18	1.44	0.72–2.86	0.30				0.65	0.44–0.96	0.032	0.65	0.43–0.97	0.034

* Surgeon and granuloma association: other surgeons compared to surgeon 3. Surgeon 5 had the lowest granuloma rate but performed substantially fewer procedures than the other surgeons. Surgeon 3 had the second lowest rate.

Notching and granuloma association: other surgeons compared to surgeon 2. Surgeon 5 had the lowest notching rate, but performed substantially fewer procedures than the other surgeons. Surgeon 2 had the second lowest rate.

### Entropion and conjunctivalization

Surgery successfully corrected entropion: 1148 (93.5%) participants at 12 months and 1126 (92.1%) at two years had no entropion, compared to 327 (25.2%) at baseline (Table 8). Surgery reduced the entropion grade in 886/918 (97%) (Paired t-test: p<0.0001). Entropion grade worsened in three participants. In the 1213 participants with conjunctivalization of the lid margin at baseline, an improvement was seen in 699 (58%) (paired t-test: p<0.0001) and worsening in 29 individuals (Fig. 1a and b).

**Table 8:** The effect of surgery on clinical characteristics at 12 and 24 months subdivided by baseline severity.

Baseline			12 months						24 months					
Clinical Feature			Minor at baseline		Major at baseline		Total		Minor at baseline		Major at baseline		Total	
Eyes	1300		620		608		1228*		613		609		1222*	
Entropion grade														
0	327	(25.2%)	598	(96.5%)	550	(90.5%)	1148	(93.5%)	588	(95.9%)	538	(88.3%)	1126	(92.1%)
1	417	(32.1%)	19	(3.1%)	34	(5.6%)	53	(4.3%)	18	(2.9%)	44	(7.2%)	62	(5.1%)
2	355	(27.3%)	3	(0.5%)	16	(2.6%)	19	(1.6%)	7	(1.1%)	22	(3.6%)	29	(2.4%)
3	122	(9.4%)	0	(0.0%)	6	(1.0%)	6	(0.5%)	0	(0.0%)	4	(0.7%)	4	(0.3%)
4	79	(6.1%)	0	(0.0%)	2	(0.3%)	2	(0.2%)	0	(0.0%)	1	(0.2%)	1	(0.1%)
Conjunctivalisation Grade														
0	9	(0.7%)	115	(18.6%)	84	(13.8%)	199	(16.2%)	74	(12.1%)	55	(9.0%)	129	(10.6%)
1	23	(1.8%)	88	(14.2%)	88	(14.5%)	176	(14.3%)	63	(10.3%)	68	(11.2%)	131	(10.7%)
2	261	(20.1%)	258	(41.6%)	249	(40.9%)	507	(41.3%)	309	(50.4%)	274	(45.0%)	583	(47.7%)
3	1005	(77.4%)	159	(25.7%)	187	(13.8%)	346	(28.2%)	167	(27.2%)	212	(34.8%)	379	(31.1%)

* Total number of participants seen at 12 and 24 months. On occasion, a specific part of the examination was not possible for a particular participant; therefore the total for each examination characteristic is not always identical to these total figures.

## Discussion

Trichiasis recurred in a quarter of study eyes by two years. Recurrence severity was very variable, ranging from a single peripheral metaplastic lash to complete entropion. However, only 13% of recurrences had more than five lashes touching they eye and there was a substantial reduction in lash burden. Therefore, although any recurrence is unsatisfactory, the likely severity of the recurrence should be considered in a balanced assessment of the risks and benefits of surgery.

Over half of all recurrences occurred by six months and the risk decreased significantly for each subsequent six-month period. Higher rates of early recurrence have been reported previously. In a study from The Gambia the recurrence at four years was 41%, over three quarters of which occurred within the first six months after surgery [22]. In a study from Southern Ethiopia, the recurrence rate at 6 weeks was 2.3% and at one year was 7.6% [17]. Taken together, these observations indicate the importance of understanding and addressing the determinants of early (<6 months) recurrence. A combination of risk factors are likely to be important: baseline disease severity, choice of procedure, the surgeon's ability, and early wound healing responses are likely to be dominant. Overall, PLTR was effective at correcting entropion, with only 8% of participants having residual entropion at the end of the follow-up. Conjunctivalization of the lid margin reversed; the epithelium with conjunctival appearance recedes and meibomion gland openings become surrounded by macroscopically normal looking skin, presumably in response to an altered epithelial environment following entropion correction.

### Pre-operative disease severity

Recurrence was twice as frequent in individuals with major TT pre-operatively. Furthermore, pre-operative entropic trichiasis (rather than misdirected or metaplastic) was an independent risk factor for recurrence. More severe pre-operative trichiasis is consistently a major risk factor for recurrent TT [8], [11], [15], [17]–[20], [22], [35], [36]. Such individuals generally have more conjunctival scarring and may have horizontal or vertical lid shortening. The lid surgery is technically more challenging as the anterior and posterior lamellae are more difficult to dissect and post-operatively there may be strong contractile forces pulling the lid back to an entropic position. These cases, who are at higher risk of sight threatening disease, should be treated by more experienced surgeons and have enhanced follow-up to detect recurrence. Interestingly, metaplastic lashes, even in the absence of entropion, appear to be ‘cured’ by surgery. It is unclear whether they cease to grow, or whether they are simply rotated far enough away from the globe.

### Surgical procedure

In our study only the PLTR procedure was used and gave recurrence rates in the middle of the reported range for this procedure: 12% to 55%, with reported follow-up periods of between 3 months and four years [8], [10], [20]–[22], [26], [37], [38]. One randomised trial has compared the PLTR and BLTR procedures and found similar outcomes [10]. However, ophthalmologists performed all the surgery, follow-up was only three months and sample size insufficient to address the question. The range of outcomes in these different studies suggests that a comparative trial of PLTR and BLTR is required under more representative operational conditions to determine if one procedure is superior, particularly for more severe cases.

### Inter-surgeon variation

The surgeons in this study were selected for their surgical ability and given additional training. Their technique was intermittently observed. Nevertheless, two surgeons had significantly higher recurrence rates than the best performing surgeon. Surgeon No. 5, who had the highest recurrence rate, did operate on a higher proportion of major TT cases than the other surgeons, but remained an independent risk factor for recurrence after adjusting for baseline TT severity.

Inter-surgeon variability has previously been highlighted as a concern in trachoma surgery with one study finding recurrence rates ranging from 0–83% between surgeons [8]. Several factors may contribute to variable outcomes. Firstly, surgical training varies in quality and number of cases performed [39]. Secondly, supervision and refresher training is often sporadic and of variable quality and content, with many surgeons operating entirely independently [39], [40]. Thirdly, surgical volume may be low which may lead to loss of surgical skills. In cataract surgery, for example, higher volume is associated with better outcomes [41]. The WHO advises that a minimum of 10 TT procedures per month should be conducted [7]. Studies from Ethiopia and Tanzania found few high volume surgeons, with the vast majority of TT surgeons perform few cases [39], [42]. In our study surgeon 5 did perform less procedures than the other surgeons as she was dismissed for disciplinary matters mid-way through the trial. However, she still conducted over 150 procedures during the trials from which this study emanates, so low surgical volume is unlikely to explain the variation. Finally, despite attempts to standardise, subtle residual variation in technical ability and technique probably remain. For example, short incisions have been associated with increased recurrence following BLTR surgery (crude OR: 3.58, 95% C.I.: 1.39–9.23) [23]. The immediate post-operative lid position warrants further investigation: if this is predictive of outcome, immediate revision could be undertaken. In programmatic settings, if individual surgeons are underperforming this needs to be addressed. Ideally, they would receive refresher training and be reassessed. Unfortunately, TT surgical audit is rarely conducted, so poor performance is probably frequently missed.

### Notching

Notching is focal external rotation or irregularity of part of the lid usually caused by excessive suture tension. Some authors include notching within a broader category of ‘lid contour abnormalities’ [11]. Large notches may cause lagophthalmos and disruption of the tear film, leading to corneal exposure. Notching can be cosmetically unsightly, in contrast to general overcorrection which is less noticeable. Other studies have reported notches in 6–30% for PLTR surgery and 0–14% in BLTR surgery [11], [21], [23], [27], [37]. The association between notching and reduced recurrence is not surprising, as notching usually reflects a degree of overcorrection. Notching occurred more frequently in older and less well nourished people (lower BMI), which may reflect age and nutrition-related reduction in tarsal plate rigidity, leading to a more pliable eyelid.

### Granuloma

Granulomas usually develop at the incision site within weeks of surgery. They require excision when they are large. In ophthalmic surgery they have been described following tarsal rotation and chalazion surgery and found to be associated with residual suture fragments, male gender and younger age [23], [43]. Here we report an association between granulomas and a lower recurrence rate and increased baseline papillary inflammation. Granulomas do not usually develop following surgery that tightly closes the incision site. In tarsal rotation surgery, the everting sutures hold the lid in an out-turned position, which may slightly part the edges of the incision from where granulomas develop. With greater external rotation, the posterior incision is less well opposed, leading to more granulation tissue formation. Granulomas may therefore be an inevitable consequence of tarsal rotation surgery with a good degree of eversion.

This study has a number of limitations that potentially constrain the generalisation of the conclusions. It is possible that the results are better than those achieved under routine operational conditions. The five surgeons were selected for their technical skill, received additional training and supervision and performed relatively large volume surgery. They are, therefore, not truly representative of many ‘field’ TT surgeons, who typically perform few cases, have limited training and supervision [39], [42]. Participants were not randomly assigned to a surgeon, however, the risk of selection bias was low, as participants were allocated on a “first-come-first-served” as surgeons became available. Finally, only one operation type, the PLTR, was used for all cases.

Set against these limitations, this study has a number of strengths. Firstly, we report the results of a large number of operations performed in a standardised manner. Secondly, follow-up rates are high despite the inaccessibility of many participants; reducing follow-up bias. Finally, participants were representative of the spectrum of TT disease in the wider population of TT patients in Ethiopia, which remains the country with the highest prevalence of TT in the world.

Recurrence rates were comparable to previous studies. Baseline disease severity and inter-surgeon variation are major determinants of recurrent disease. However, PLTR surgery successfully corrected most entropion and much of the recurrence was minor, which may not represent a significant risk for most patients. The inter-surgeon variation in recurrence rates is concerning. Further research is needed to ascertain whether recurrence can be predicted immediately after surgery, and whether it can be ameliorated.

## Supporting Information

Checklist S1 STROBE Checklist.
(DOC)
